# Octyl gallate induces hepatic steatosis in HepG2 cells through the regulation of SREBP-1c and PPAR-gamma gene expression

**DOI:** 10.17179/excli2020-2214

**Published:** 2020-07-06

**Authors:** Kelly Goulart Lima, Vitor Giancarlo Schneider Levorse, Maria Claudia Rosa Garcia, Bruno de Souza Basso, Bruna Pasqualotto Costa, Gessica Luana Antunes, Carolina Luft, Gabriela Viegas Haute, Léder Leal Xavier, Márcio Vinícius Fagundes Donadio, Jarbas Rodrigues de Oliveira

**Affiliations:** 1 Laboratório de Biofísica Celular e Inflamação, Escola de Ciências da Saúde e da Vida, Pontifícia Universidade Católica do Rio Grande do Sul (PUCRS), Brazil; 2 Laboratório de Biologia Celular e Tecidual, Escola de Ciências da Saúde e da Vida, Pontifícia Universidade Católica do Rio Grande do Sul (PUCRS), Brazil

**Keywords:** hepatic steatosis, octyl gallate, HepG2 cells, lipid droplet, SREBP-1c, PPAR-gamma

## Abstract

Octyl gallate (OG) is an antioxidant commonly used in food, although there is no definition of its acceptable daily intake. There are reports *in vitro *and *in vivo* showing that food additives and drugs can alter lipid metabolism. Lipid droplet accumulation in hepatic cells is one of the main findings in the unregulated lipid metabolism and is strongly related to the development of nonalcoholic fatty liver disease (NAFLD). In this study, we investigated the effects of OG on lipid metabolism in the hepatocellular carcinoma cell line (HepG2). The results have shown, for the first time, that treatment with OG increased the overall amount of lipids, the triglyceride concentration, the lipid droplet area, and SREBP-1c and PPAR-γ gene expression. Taken together, the findings indicate that OG induces lipid droplet accumulation in HepG2 cells through the regulation of SREBP-1c and PPAR-γ gene expression without involving mTOR/S6K1 and may contribute to NAFLD when used as a food additive.

## Introduction

Hepatic steatosis is defined as the presence of intrahepatic fat of at least 5 % of the liver weight (Nassir et al., 2015[24]). Non-alcoholic fatty liver disease (NAFLD) includes both simple and progressive steatosis with associated hepatitis (steatohepatitis), fibrosis, cirrhosis, and eventually hepatocellular carcinoma (Sayiner et al., 2016[28]). There are reports stating that the use of drugs such as tamoxifen (Zhao et al., 2014[36]), glucocorticoids (Letteron et al., 1997[20]) and 5-fluorouracil (Gentilucci et al., 2006[14]) may lead to NAFLD. Furthermore, several studies have shown that food additives may also induce NAFLD, including fructose (Chen et al., 2017[3]), monosodium glutamate and aspartame (Collison et al., 2013[4]). 

Octyl gallate (OG) is an antioxidant commonly used in food, although there is no current definition of its acceptable daily intake (ADI), considering that studies on its pharmacokinetics and metabolism are scarce (World Health Organization, 1997). In the United States, OG is approved only as a margarine additive in a concentration of 0.0075 % or less (U.S. Food and Drug Administration, 2018[31]), while in Europe it is authorized as a food additive in heat-treated processed meat, chewing gum and processed potato products (European Parliament and the Council of the European Union, 2008[12]; European Food Safety Authority, 2015[11]). The most recent European re-evaluation regarding the use of OG concluded that, considering the lack of studies on its pharmacokinetics and metabolism, an adequate assessment of its safety as a food additive is needed (European Food Safety Authority, 2015[11]).

An animal model study showed that OG, at high doses, was able to induce strong inhibition of gluconeogenesis, which could be considered as harmful (Eler et al., 2015[10]). In addition, OG has been shown to induce toxicity in mice liver, as its use increased serum levels of AST and ALT, oxidative stress and histological damage (Cordova et al., 2017[5]). However, no studies have evaluated its effect on lipid metabolism. Thus, considering that the human hepatocellular carcinoma cell line (HepG2) is widely used as an *in vitro* model to study steatosis, as it accumulates lipids in the cytoplasm similarly to hepatocytes (Cui et al., 2010[6]; Alkhatatbeh et al., 2016[1]; Zeng et al., 2016[35]), the aim of this study was to investigate the effects of OG on lipid metabolism in HepG2 cells.

## Materials and Methods

### Cell culture and treatment

HepG2 cells (Banco de Células do Rio de Janeiro – BCRJ, code: 0103) were cultivated in Dulbecco's Modified Eagle Medium (DMEM; Gibco, Life-technologies, USA), containing fetal bovine serum (FBS, 10 %; Gibco, Life-technologies, USA) and 1 % penicillin/streptomycin antibiotics (ATB; Gibco, Life-technologies, USA). The cells were incubated in a humidified atmosphere at 37 °C with 5 % CO_2_. The OG stock solutions were prepared in dimethylsulfoxide (DMSO; Neon, Brazil) and the dilution was prepared in DMEM containing 10 % FBS. Oleic acid (OA; Synth, Brazil) was used as a positive control to induce lipid droplet (LD) accumulation in HepG2 cells (Cui et al., 2010[6]). The OA stock solutions were prepared in isopropanol and the dilution was prepared in DMEM containing 10 % FBS. The control cells were maintained in DMEM, 10 % FBS and DMSO 0.2 % (vehicle). The treated cells received OG at 40 µM and the positive control cells received OA at 100 µM. The HepG2 cells were incubated and the analyses were performed 24 h after treatment.

### Morphological analysis

HepG2 cells (12 x 10^4^ cells/well) were seeded into 24-well plates, cultured, and treated as aforementioned. Cellular morphological changes were evaluated after 24 h of treatment using a phase contrast inverted microscope (INV100, BEL Engineering, Italy) at 400× magnification and photographed using a Bel Photonics camera. 

### Oil Red Staining

Cells were seeded as described for the morphological analysis and treated as aforementioned. After the treatment period, the culture medium was removed and HepG2 cells were washed twice with a phosphate-buffered saline (PBS) solution. Afterwards, HepG2 cells were fixed for 30 min with 4 % paraformaldehyde solution, then washed thrice with PBS, and subsequently rinsed with propylene glycol. Then, cells were stained with freshly prepared propylene glycol solution containing 0.5 % Oil Red (Sigma-Aldrich, USA), previously filtered with a 0.45 µm filter, for 15 min. Thus, cells were rinsed with 60 % propylene glycol and washed with distilled water. Images of cells with the stained lipid droplets were acquired using a phase contrast inverted microscope (INV100; BEL Engineering, Italy) at 400× magnification and photographed using a Bel Photonics camera.

### Lipid measurement

After the Oil Red (Sigma-Aldrich, USA) stained lipid droplet images were acquired, 200 µL of isopropanol was added to each plate well for the lipid dissolution. Optical density (OD) was monitored with a VICTOR® microplate reader (PerkinElmer) at a wavelength of 490nm. Cell viability was evaluated using the Trypan blue (Sigma-Aldrich, USA) exclusion assay, as previously described (Lima et al., 2018[22]), in order to correct the optical density lipid reading by the number of cells. Results were expressed as Lipid OD/100,000 cells.

### Triglyceride assay

Cells were seeded into 60.8 cm^2^ plates (5 x 10^6^ cells/well), cultured and treated as aforementioned. Then, HepG2 cells were transferred to 1.5 mL tubes and all samples were centrifuged at 3000rpm for 5min. The pellet was washed once using PBS, resuspended with 400 μL of PBS solution, and submitted to ultrasonication. The cellular triglyceride (TG) concentration was measured through TG Color assay kit (Wiener lab, Argentina). All measurements were normalized by protein concentration, following the protocol recommended by the manufacturer. Absorbance was measured at 505 nm using CT300i Chemistry Analyzer (Wiener lab, Argentina).

### Transmission Electron Microscopy (TEM) analysis

Initially, a trypsinization was performed for the collection of semi-confluent HepG2 cells. Then, the cells were prepared as previously described (Lima et al., 2018[22]). For the ultrastructural analysis in both control (DMSO 0.2 %) and treated cells (OG 40 µM), a transmission electron microscopy (FEI Tecnai T20 G2) and the Image Pro Plus software (Image Pro Plus 6.1, Media Cybernetics, Silver Spring, EUA) were used. All the analyses were performed by two blinded specialists in imaging analysis. The TEM images were used for morphological evaluation (lipid droplet density and area).

### Lipid droplet density

In order to estimate lipid droplet density, a quantitative analysis was performed. For that, randomized squares (3.0 μm^2^), named as areas of interest (AOIs), were overlaid on each image captured (4500×) and the lipid droplet located inside each square, as well as in the edges (upper and/or left), were counted. On the other hand, lipid droplet located in the lower and/or right edges of the squares were not counted (de Senna et al., 2017[8]). In average, 7 AOIs were evaluated in each cell and a total of 10 cells were evaluated per group.

### Lipid droplet area

The lipid droplet area was calculated using a stereological tool and the point counting method (Weibel 1979[32]; Zacharová and Kubínová, 1995[34]; Ilha et al., 2008[15]; Lazzarotto Rucatti et al., 2015[19]; Fernandes et al., 2016[13]). A grid mask containing an area/point value of 6518.5 nm^2^ was placed over the lipid droplet image (13500×). Each point of the grid has a cross shape, thus delimiting four quadrants. The lipid droplets that were superimposed on the upper right quadrant of the grid points were counted. The areas were obtained through the following equation: Â = ∑p.a/p (Â is the area and ∑p is the sum of points counted, and a/p is the area/point value). In average, 5 lipid droplets were evaluated in each cell and a total of 6 cells were evaluated per group.

### Western blot analysis 

HepG2 cells (5 x 10^5^ cells/well) were seeded in 6-well plates, cultured and treated as aforementioned. The preparation of the cells was performed as previously described (Lima et al., 2018[22]). Protein samples were separated with sodium dodecyl sulphate polyacrylamide gel electrophoresis (SDS-PAGE) and transferred to the nitrocellulose membrane. Next, the blot was blocked for 1 hour with Tris-buffered saline containing 0.05 % Tween-20 and 5 % non-fat dry milk. Then, the blot was incubated overnight at 4 °C with the following primary antibodies: mTOR (1:500) (2972, Cell Signaling) and GAPDH (1:1000) (39-8600, Invitrogen). After incubation with primary antibodies, the blot was incubated again for 1 hour with the secondary antibody conjugated with horseradish peroxidase (HRP) (1:1000). The chemiluminescence and digital images were taken in a Carestream Gel Logic 2200 PRO Imaging System. The protein concentration was measured using the NanoDrop Lite (Thermo Fisher Scientific®). GAPDH was used to normalize protein quantification (de Mesquita et al., 2017[7]).

### RNA isolation and quantitative PCR

HepG2 cells (2.5 x 10^5^ cells/well) were seeded in 6-well plates, cultured and treated as aforementioned. The RNA of all cells was extracted using TRIzol® Reagent (Invitrogen), according to the manufacturer’s recommendations. Then, RNA was converted to complementary deoxyribonucleic acid (cDNA) using Superscript III First-Strand Synthesis SuperMix (Invitrogen), according to the manufacturer’s instructions. Primer sequences used were synthesized by Integrated DNA Technologies (Iowa, USA) and the sequences are described in Table 1(Tab. 1). The quantity of cDNA was evaluated using the NanoDrop 2000 (Thermo Fisher Scientific). The gene expression of SREBP1c, PPAR-α, PPAR-γ, and β-actin were quantified using StepOneTM (Applied Biosystems). The reaction was catalyzed using the SYBR Green I (Applied Biosystems - Thermo Fisher Scientific), according to the manufacturer’s recommendation. The β-actin was used as an endogenous control gene.

### Data and statistical analysis

The results from three independent experiments are presented as mean ± standard deviation (SD). The Student’s t-test was used for comparison between groups in the Ultrastructural analysis (TEM), Western blotting, and quantitative PCR assays. For the comparisons between groups in the Lipid Measurement and TG assays, a one-way analysis of variance (ANOVA), followed by Tukey post hoc test, was used. In all cases the significance level was set at *p*<0.05. Statistical analyses were performed using GraphPad Prism 5.0 (GraphPad Software, San Diego, CA).

## Results

### Octyl gallate induces lipid accumulation in HepG2 cells

**Figure 1:**
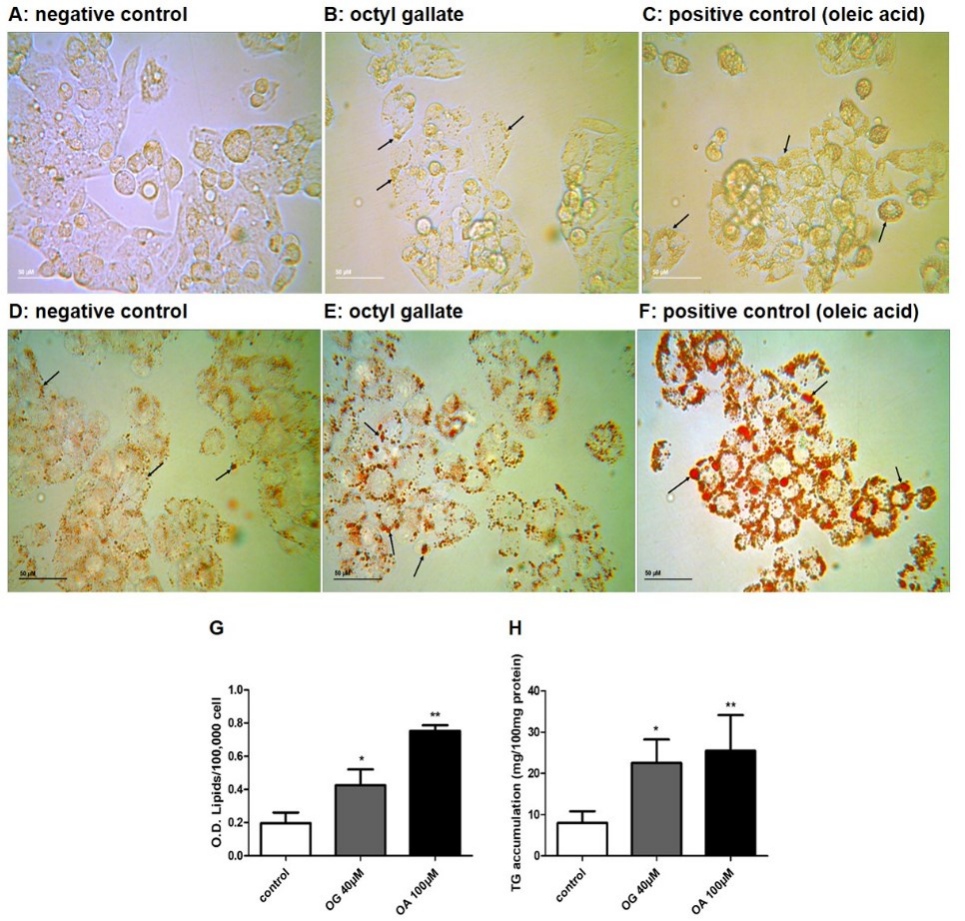
Effects of OG on HepG2 cell morphology after 24 h. Representative images of control cells (A), OG-treated cells (B) and OA-treated cells (C) are presented. Black arrows show lipid droplets. Effects of OG on steatosis induction in HepG2 cells determined by Oil Red staining and TG assay. Representative images of control cells (D), OG-treated cells (E) and OA-treated cells (F) are presented. Results are expressed as Optical density/1×10^5^ cells (G) and TG accumulation (mg/100 mg protein) (H). Data are represented as the mean + SD from three independent experiments (*p<0.05, **p<0.01). Treatment with OG induced lipid droplet accumulation in the cell cytoplasm (E). OA led to lipid droplet accumulation with greater intensity (F). Treatment with OG increased overall amount of lipids (G) and TG concentration (F). OA: oleic acid. Calibration bar = 50 µm.

Treatment with OG induced vesicle accumulation in the cell cytoplasm, as observed in the phase contrast inverted microscope (Figure 1B(Fig. 1)). The positive control for the induction of lipid droplet accumulation (treatment with oleic acid) has also led to cytoplasmic vesicle accumulation, although with a greater intensity, as shown in Figure 1C(Fig. 1). Oil Red staining assay revealed that treatment with OG significantly increased lipid accumulation, as shown in Figure 1G(Fig. 1) (representative image shown in Figure 1E(Fig. 1)). As expected, the positive control increased lipid accumulation in a greater intensity (Figure 1F and Figure 1G(Fig. 1)). The results also showed an increase in the TG concentration after OG treatment (Figure 1H(Fig. 1)), corroborating the accumulation of lipids demonstrated in the Oil Red assay.

### Ultrastructural analysis showed that OG increases lipid droplet area without modifying lipid droplet density

**Figure 2:**
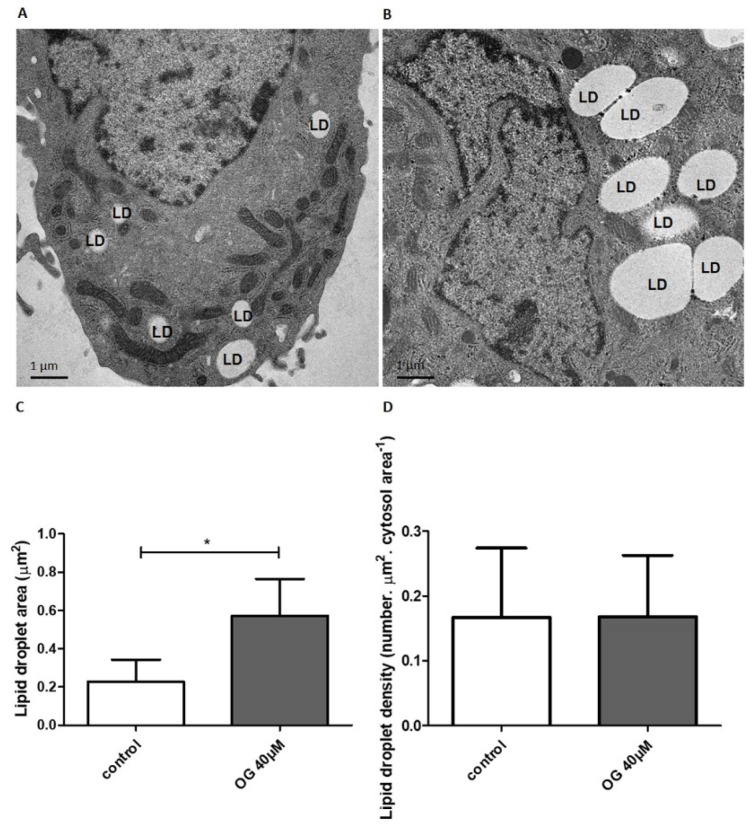
Ultrastructural analysis of HepG2. Representative images of control cells (A) and OG-treated cells (B) are presented. LD: lipid droplet. Calibration bar = 1 µm. TEM images revealed that treatment with OG increased LD area (C) without changing LD density (D). Results are expressed as LD area in µm^2 ^(C) and number of LD per µm^2^ of cytosol area (D). Data are represented as the mean + SD from three independent experiments (*p<0.05).

In order to confirm the effects of OG on lipid accumulation and to evaluate lipid droplet area and density, an ultrastructural analysis of HepG2 cells was performed. Representative images are shown in Figure 2A(Fig. 2) (control) and Figure 2B(Fig. 2) (40 µM OG). Transmission electron microscopy images revealed that treatment with 40 µM of OG increased the lipid droplet area (*p*<0.05; Figure 2C(Fig. 2)) without changing lipid droplet density (*p*=0.97; Figure 2D(Fig. 2)). 

### Octyl gallate decreases mTOR protein expression 

**Figure 3:**
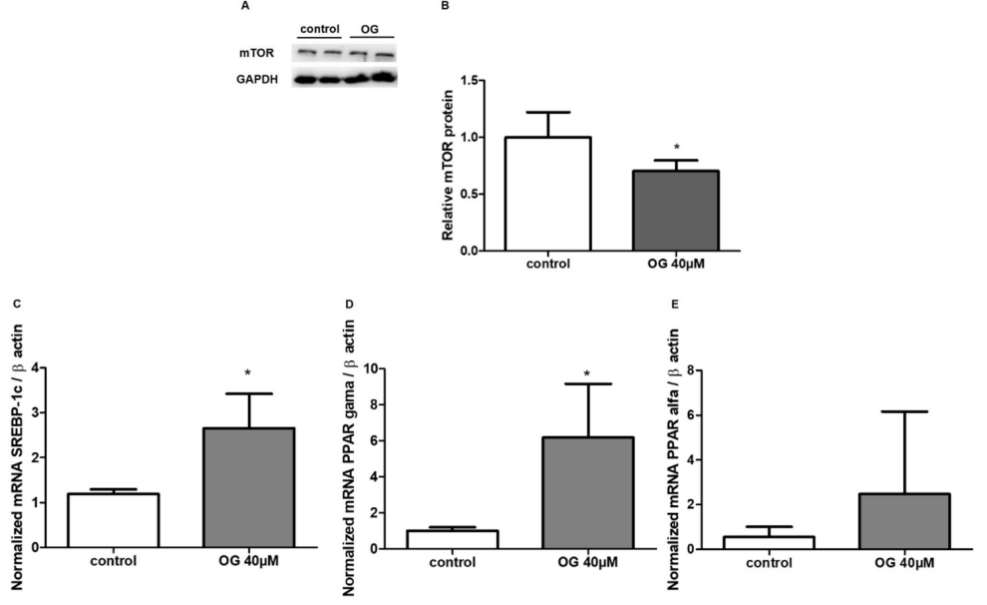
Expression of mTOR protein in HepG2 cells after 24 h of treatment with OG by Western blot analysis (A). GAPDH was used to normalize protein expression. Results are expressed as protein levels (fold induction) of the mTOR expression. Treatment with OG decreased the expression of mTOR (B) in HepG2 cells (*p<0.05). In C, D and E the effect of OG on mRNA expression of SREBP-1c (*p<0.05), PPAR-γ (*p<0.05) and PPAR-α (p=0.42) genes regulating lipid metabolism in HepG2 cells is shown. Results are expressed as relative expression rate. All data are represented as the mean ± SD of 3 independent experiments.

In order to investigate whether increased mammalian target of rapamycin (mTOR) expression could be related to lipid accumulation caused by OG, we have evaluated its expression using Western blot. However, results have shown that treatment with OG decreased mTOR expression (*p*<0.05; Figure 3A and B(Fig. 3)).

### Octyl gallate increases SREBP-1c gene expression 

Therefore, the involvement of mTOR/ SREBP-1c signaling in lipid accumulation was investigated by measuring the SREBP-1c gene expression. Results have shown that treatment with OG increased SREBP-1c gene expression (*p*<0.05; Figure 3C(Fig. 3)), although a reduction in the mTOR protein expression was seen.

### Octyl gallate increases PPAR-γ gene expression

Finally, we have investigated whether the modification of PPAR-γ and PPAR-α gene expression could be related to lipid accumulation induced by OG, by evaluating the expression of its genes. Results have shown that OG increased PPAR-γ gene expression (*p*<0.05; Figure 3D(Fig. 3)) without modifying PPAR-α (Figure 3E(Fig. 3)).

## Discussion

The initial toxicity studies performed with OG aimed to determine the acceptable daily intake (ADI) in order to regulate the use of this antioxidant as an additive in food. Thus, at that time, an ADI for man of 0.2 mg/kg body weight (as a sum of propyl, octyl and dodecyl gallates) was defined (Joint FAO/ WHO Expert Committee, 1966[18]). Subsequent studies in rats, evaluating the effects of OG, showed high mortality levels in young pups. In addition, there were observations that OG caused reactions of sensitization in the oral mucosa of individuals previously sensitized by cutaneous contact with OG (Joint FAO/ WHO Expert Committee, 1974[17]). 

In a re-evaluation of the committee in 1997, a decision that it would not be possible to maintain the definition of the ADI for the octyl and dodecyl gallates was taken, considering the lack of studies on pharmacokinetics and metabolism (World Health Organization, 1997). Ever since, there is no definition of the ADI for OG. Several countries have used the OG as an additive in certain foods, although restricted to specific foods and with its maximum acceptable concentration determined for each authorized type of food (European Parliament and the Council of the European Union, 2008[12]; European Food Safety Authority, 2015[11]; U.S. Food and Drug Administration, 2018[31]). Nevertheless, to date, there are no studies in the literature showing the effects of OG on hepatic lipid metabolism.

The concentration used to treat HepG2 cells in the present study was 11.29 mg/L (40 μM), which is equivalent to the estimated plasma concentration after a daily consumption of 72.2 g of products containing the maximum concentration of OG (400 mg per kg or liter of food) (European Food Safety Authority, 2015[11]). The present results have shown, for the first time, that OG has the ability to induce lipid accumulation in an *in vitro* model of hepatic steatosis. Studies with other food additives (Collison et al., 2013[4]; Chen et al., 2017[3]; Jensen et al., 2018[16]) or therapeutic drugs (Gentilucci et al., 2006[14]; Menter et al., 2009[23]; Zhao et al., 2014[36]) have also found lipid accumulation in the liver. Our results are also in agreement with an *in vivo* study showing that OG induced liver toxicity, as its treatment increased the oxidative stress (Cordova et al., 2017[5]). Considering that the excess of free fatty acids increases the production of reactive oxygen species (Svegliati-Baroni et al., 2019[30]), it is possible that these mechanisms are associated.

Considering that the regulation of lipid metabolism via mTOR/SREBP-1c signaling is well known (Porstmann et al., 2008[26]; Li et al., 2015[21]), we have investigated whether the mechanism of lipid accumulation induction by OG could be related to increased mTOR expression. Interestingly, results have shown a reduction in the expression of this protein, which is in agreement with our previous study showing a reduction of cellular growth (Lima et al., 2018[22]). 

The transcription factor SREBP-1c is the major isoform that controls fatty acid synthesis in the liver (Pettinelli et al., 2011[25]), as it promotes transcription of lipogenic genes (Sanders and Griffin, 2016[27]). Our findings showed that OG up-regulated SREBP-1c gene, similarly to previous studies with tamoxifen (Zhao et al., 2014[36]) and fructose (Shrestha et al., 2009[29]). Considering that the regulation of the transcription factor SREBP-1C may occur through both mTOR/S6K1 (Düvel et al., 2010[9]) or an independent pathway (Yap et al., 2011[33]), it is plausible to consider that the OG induced lipogenesis may involve the regulation of the SREBP-1C gene without the involvement of mTOR/S6K1. Thus, other signaling pathways may be involved in the effects of OG, such as signaling via Liver X receptors (LXR).

The peroxisome proliferator-activated receptors (PPARs) act mainly in the regulation of metabolic pathways related to fatty-acid oxidation and lipid metabolism (Ament et al., 2012[2]). PPAR-α regulates a number of catabolic pathways and PPAR-γ appears to be key in lipid storage and synthesis, as well as in adipogenesis. Thus, we have investigated if changes in the PPAR-α and PPAR-γ expression could be related to lipid accumulation induced by OG. Results have shown that OG increased, at approximately 6-fold, the PPAR-γ gene expression, without modifying the PPAR-α. Thus, lipogenesis caused by OG involves the regulation of the two major transcription factors that control lipid storage and synthesis, PPAR-γ and SREBP-1c. These results are in agreement with a previous study showing that, in the liver of obese patients with NAFLD, the PPAR-γ is up-regulated, demonstrating an additional lipogenic mechanism to SREBP-1c induction on the development of hepatic steatosis (Pettinelli et al., 2011[25]). 

In conclusion, treatment with OG increased the overall amount of lipids, the triglyceride concentration and lipid droplet area. The results presented here show, for the first time, that OG induces lipid droplet accumulation in HepG2 cells through the regulation of SREBP-1c and PPAR-γ gene expression, without involving mTOR/S6K1. Taken together, data suggests that the use of maximum OG concentration as a food additive may lead to NAFLD.

## Acknowledgements

The authors would like to thank their colleagues from the Immunodiagnostic Laboratory and the Central Laboratory of Microscopy and Microanalysis (LabCEMM) at PUCRS. 

## Funding

This study was financed in part by the Coordenação de Aperfeiçoamento de Pessoal de Nível Superior – Brasil (CAPES) – Finance Code 001, and Conselho Nacional de Desenvolvimento Científico e Tecnológico (CNPq).

## Conflict of interest statement

The authors declare no conflict of interests.

## Figures and Tables

**Table 1:**
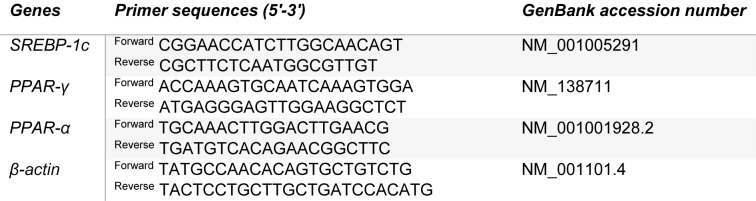
Primer sequences used for qRT-PCR
